# Tumor specificity of WNT ligands and receptors reveals universal squamous cell carcinoma oncogenes

**DOI:** 10.1186/s12885-022-09898-2

**Published:** 2022-07-19

**Authors:** Cheng Chen, Lunan Luo, Changling Xu, Xia Yang, Ting Liu, Jingyue Luo, Wen Shi, Lu Yang, Yi Zheng, Jing Yang

**Affiliations:** 1 Department of Health Management, Sichuan Provincial People’s Hospital, University of Electronic Science and Technology of China, Chengdu, 610054 China; 2 School of Medicine, University of Electronic Science and Technology of China, Chengdu, 610054 China; 3 Department of Oral Medicine, Peking University School and Hospital of Stomatology, Beijing, 100191 China

**Keywords:** WNT signaling, Tumor specificity, SCC, WNT5A, WNT7B

## Abstract

**Background:**

The WNT signal pathway has myriad family members, which are broadly involved in embryonic development and human cancer. Over-activation of WNT-β-Catenin signaling promotes cancer cell proliferation and survival. However, how diverse components of WNT signaling specifically engaged in distinct tumor types remains incompletely understood.

**Methods:**

We analyzed the transcriptomic profiling of WNT ligands and receptors/co-receptors among 26 different tumor types to identify their expression pattern, and further verified these results using clinical oral squamous cell carcinoma (OSCC) and lung squamous cell carcinoma (LUSC) samples. At the same time, we also detected WNT7B expression in oral inflammation and carcinoma, and constructed stable WNT7B knockdown OSCC cell lines to study the effects of WNT7B on the cell migration and invasion ability.

**Results:**

We found a group of tumor-specific WNT members, including a panel of squamous cell carcinomas (SCCs) specific upregulated WNT ligands and receptors, WNT5A, WNT7B, FZD7 and GPC1. We further revealed a significant correlation between these protein expression characteristics and clinical outcomes of OSCC and LUSC patients. Moreover, WNT7B was demonstrated to contribute to the development of oral chronic inflammation and OSCC, partly due to promoting the invasion ability of tumor cells.

**Conclusions:**

These results demonstrate that the function of WNT ligands and receptors in specific tumors depends on the origination of tumor tissue type. Collectively, they support the use of WNT components as a highly specific target for pan-tissue-type originated tumors.

**Supplementary Information:**

The online version contains supplementary material available at 10.1186/s12885-022-09898-2.

## Introduction

WNT signaling represents an evolutionarily conserved pathway controlling embryonic development and adult homeostasis [1, 2]. WNT ligands comprise a family of 19 secreted glycoproteins, which bind to more than 15 WNT receptors and co-receptors. Different combinational types between WNT components determine the distinct downstream pathways directing cell fate [3]. WNT signaling could be classified into three pathways, including WNT/β-catenin, WNT/PCP and WNT/Ca^2+^ pathway. WNT/PCP pathway participated in cell polarity and migration, whereas WNT/Ca^2+^ pathway is crucial for cell adhesion and motility during gastrulation [3]. As for canonical WNT/β-catenin signaling, WNTs bind to receptors and co-receptors, which recruit Dishevelled and subsequently interact with the β-catenin destruction complex to regulate the turnover of cytosolic β-catenin, thus accumulating the nucleus β-catenin and activating a gene transcriptional program via TCF/LEF-1, consequently altering cellular processes such as proliferation, differentiation and stemness [2].

However, dysregulation of WNT signaling has been intimately associated with human diseases such as cancers and inflammation [2, 4]. Aberrant WNT/β-catenin signaling may contribute to solid tumors and leukemia [5]. Moreover, growing lists of loss-of-function mutations in WNT tumor suppressor genes are observed in carcinomas including colorectal cancer and OSCC [6–8]. WNT ligands also changed expression levels in cancers, e.g. WNT3A, WNT5A and WNT7B were upregulated in cholangiocarcinoma [9]. Extensive efforts have been made to block WNT signaling in cancer with small molecules or monoclonal antibodies. For example, numerous drug candidates targeting FZD or Porcupine, an acyltransferase enabling specifically secretion of all WNT ligands, have been developed [10]. Mechanically, pancreatic tumor cells were attenuated growth by antibodies directed at FZDs [11]. So far, a variety of monoclonal antibodies targeting human FZD receptors is undergoing clinical trials, for instance, OMP-18R5 for breast and pancreatic cancer, OTSA101 for advanced synovial sarcoma [4, 12].

Despite major WNT pathway components having been well uncovered, the function of WNT signaling within tumorigenesis is highly intricate and remains unclear. The expression pattern of WNT ligands and receptors is distinct from each other. Consequently, WNT ligand-receptor interaction is extremely promiscuous. A single WNT can bind to distinctive FZD receptors, and vice versa, thus there exist extensive cross-reactivity between WNT ligand and receptor [3]. It raised issues of WNT signaling specificity: do individual WNT or WNT/receptor interactions have a unique or overlapping function? Do WNT components involved in cancer have tumor specificity?

To address these questions, we comprehensively characterized the expression profiling of WNT ligands and receptors/co-receptors among 26 kinds of tumors and dissected the role of several WNTs in SCC development. Taken together, these results suggest that expression of WNT ligands and receptors in specific tumors depends on the tissue type where they originate in the body and WNT components may assist the transition of inflammation to cancer, which could be utilized to develop biomarkers for tumor diagnosis and therapeutic strategy for targeting pan-tissue type originated tumors.

## Materials and methods

### Database for mRNA sequencing data analysis

Two RNA sequencing datasets were used for analysis in this work. One was our previously published OSCC and OLP dataset (GSE70666; http://www.ncbi.nlm.nih.gov/geo/query/acc.cgi?acc=GSE70666). Another dataset was downloaded from Expression Atlas, which is an open science resource offering information about gene and protein expression across species and biological conditions (https://www.ebi.ac.uk/gxa/experiments/E-MTAB-5200), where we downloaded data of 26 kinds of tumors with their adjacent normal tissues. All data used for analysis were provided in Supplementary data.

### Patients and samples

This study was approved by the Peking University Institutional Review Board with written permission (No. IRB00001052–12037), written informed consents were obtained from patients for research using tissue samples. All OSCC and OLP patient samples were obtained from School of Stomatology, Peking University Health Science Center (PKUHSC), Beijing, China. LUSC patient tissue chips were purchased from Shanghai Outdo Biotech Co., Ltd.

For qRT-PCR validation of WNT5A, WNT7B, WNT2B and WNT10A, paired tumor specimens and adjacent normal tissues were derived from 21 primary OSCC patients, who received curative surgery from 2008 to 2013. Fresh frozen tissues were preserved in liquid nitrogen until use. An additional set of 34 FFPE samples was used for IHC validation, collected between 2008 and 2013, 15 were OLP tissues and others were paired OSCC and adjacent normal samples. 4 sets of LUSC tissue chips were used to verify WNT5A, WNT7B, FZD7 and GPC1 protein expression in paired tumor specimens and adjacent normal tissues, the patients received surgery from 2007 to 2009, and got the latest follow-up in July 2012. Clinical parameters, including age, sex, pathological features, and TMN stage, were retrospectively collected by reviewing patients’ charts (Supplementary Table 1).

### Cell lines and antibodies

The human embryonic kidney 293 T (HEK 293 T) and human OSCC cell lines, including FaDu and SCC9, were obtained from the National Platform of Experimental Cell Resources for SCI-Tech (Beijing, China). All cell lines were maintained in Dulbecco’s Modified Eagle’s Medium (DMEM; Gibco, NY, USA), supplemented with 10% fetal bovine serum (FBS; Gibco), 100 U/ml penicillin and 100 μg/ml streptomycin (Macgene, China). Cells were cultured at 37 °C in a humidified incubator under 5% CO_2_. Primary antibodies used for immunohistochemistry detection and Western blot were as follows: WNT7B (Abcam), WNT5A (Abcam), FZD7 (Abcam) and MMP1 (Abcam), GPC1 (Sigma-Aldrich). β-Tubulin and GAPDH antibodies used for Western blot were obtained from Solarbio Science & Technology Co., Ltd.

### RNA extraction, reverse transcription and real-time qPCR

Total RNA was extracted using a TRIzol reagent (Invitrogen, #15596026). For each extract, aliquots of total RNA (1 μg) were used for first-strand cDNA synthesis with random hexamer primer using the Revert Aid First Strand cDNA Synthesis Kit (Thermo Fisher Scientific, #K1621). Supplementary Table 2 shows the primers used in this study. Real-time qPCR was then carried according to the guidelines (Applied Biosystems) employing a 7500 Fast Real-Time PCR System, following the protocol for the 2 × ChamQ Universal SYBR qPCR Master Mix (Vazyme, #Q711–02). Transcript levels were calculated relative to β-Actin (clinical patient tissues) or GAPDH (cell lines), and the relative expression of each mRNA was calculated using the values of 2^-ΔΔCT^. Each measurement was performed in technical triplicates.

### Construction of stable *WNT7B* knockdown cell lines

Three shRNA interference sequences and a control sequence were chosen (Supplementary Table 3) to construct stable WNT7B knockdown cell lines. For lentivirus production, HEK 293 T cells were plated into a 6-well plate and co-transfected with 2.5 μg shRNA lentiviral vector (pLV-shRNA-EGFP) carrying different WNT7B shRNA sequences, 1.5 μg Δ8.9 plasmid and 1 μg VSV-G plasmid using Megatran1.0 (OriGene, #TT210002) according to the manufacturer’s instructions. The supernatant was collected 48 hours and 72 hours post-transfection and filtered with 0.45 μm filters. For stable cell lines generation, SCC9 and FaDu cells were infected with different shRNA virus in 24-well plates separately, supplemented with 8 μg/ml polybrene (Solarbio, # H8761) per well, and then selected with 0.5 μg/ml puromycin (Solarbio, #P8230) for 14 days.

### Cell proliferation assays

The effect of WNT7B knockdown on SCC9 and FaDu cells was assessed using the Cell Counting Kit-8 (CCK-8; Dojindo, #CK04–11). Briefly, the cells were seeded into a 96-well plate at 2 × 10^3^ cells per well, and 10 μl CCK-8 reagent was added to each well at various time points, then incubated at 37 °C for 2 hours. The absorbance was measured at 450 nm. Triplicate wells were measured for cell viability in each group, and 4-time points (0, 24, 48, 72 hours) were chosen to measure.

### Transwell migration and invasion assay

Cells were grown in serum-free media for 24 hours before being placed on the upper layer of a permeable membrane-coated cell culture insert. Below the cell permeable barrier was placed complete growth media with 15% FBS as an attractant. Following an incubation period, the cells that had migrated through the membrane were stained with crystal violet and quantitated using ImageJ software. The invasion assay was carried out in the same manner as the above, with the exception that the cell culture insert was covered with matrigel.

### Western blot analysis

Whole-cell extracts were obtained with RIPA buffer (Solarbio, # R0020) supplemented with PMSF and protease inhibitors. Lysates were briefly sonicated and centrifuged at 12000 × g at 4 °C for 20 min. The protein amount was quantified using BCA protein assay (Solarbio, # PC0020) according to the manufacturer’s instructions. A total of 20 μg protein was loaded onto and electrophoresed in an SDS-PAGE gel and then transferred onto polyvinylidene difluoride (PVDF) membranes (Millipore). After being blocked with 5% Difco Skim Milk (BD Pharmingen, #232100) in TBST solution, the membrane was incubated with primary antibodies at 4 °C overnight. After washing, membranes were further incubated for 60 min at room temperature with the secondary antibodies. The signals were detected with Immobilon Western Chemiluminescent HRP Substrate (Millipore, #WBKLS0100). Finally, the membrane was scanned with an Automatic Chemiluminescence Image Analysis System (Tanon).

### Cell cycle detection by flow cytometer

Cells were plated in six-well plates, followed by incubation with serum-free medium for 48 hours for cell cycle synchronization, and then recovered serum. Cells were collected at 2-time points, 0 and 24 hours, after serum recovery. The attached cells were harvested and washed with 1 ml PBS, then centrifuged at 1000 rpm for 10 min and discarded the supernatant. Fixed cells with 1 ml pre-cooled 75% ethanol, vortexed, and kept cells at 4 °C overnight. Then centrifuged cells at 1000 rpm for 10 min and discard the supernatant. Stained cells with cell cycle detection kit (BD Pharmingen, #554656) after washed with PBS, resuspend the cells in 0.5 ml PT/RNase staining solution (BD Pharmingen, #550825), 1 × 10^6^ cells per tube, and incubated at room temperature in the dark for 15 min, then detect with NovoCyte Flow Cytometer (ACEA Biosciences).

### Immunohistochemistry and analysis

Formalin-fixed and paraffin-embedded tissues were cut into 5 μm slices. Slices or tissue chips were deparaffinized in xylene, rehydrated in a graded ethanol concentration (100, 95, 80 and 70%) and then submerged in PBS. The slices were blocked endogenous peroxidase with 3% hydrogen peroxide solution for 20 min and placed in an autoclave with 0.01 M sodium citrate solution at 120 °C for 3 min for antigen retrieval. Slices were incubated with primary antibody overnight at 4 °C, then detected using the Enhanced Polymer Detection System for Immunohistochemical Staining kit (Zhongshan Golden Bridge, # PV-9000), and DAB was used as a chromogen. Negative controls were performed by replacing the primary antibody with PBS. All slices were then counterstained with hematoxylin and then observed and captured in a bright field microscope.

A scoring method was used to evaluate protein expression. The immunostaining was reviewed by two independent evaluators. Immunohistochemical (IHC) reactivity was graded according to the percentage of different intensity positive tumor cells (a) and the intensity of staining (b): (0) no staining, (1) suspected positive, (2) weak, (3) moderate, and (4) intense staining compared to the negative control. The final IHC score (c = a × b) was a weighted score calculated for each specimen. The stained tissues were scored blindly without knowing clinical patient data.

### Statistical analysis

A non-parametric Mann-Whitney U test was used to analyze the relationship between the qRT-PCR numerical values of two groups. Paired or unpaired Student’s t-tests were used for tissues and in vitro experiments. Linear regression was used to analyze the correlation of WNT7B and MMP1 protein expression in tissues. Statistical analyses were performed using GraphPad Prism v7.0 software (GraphPad Software Inc., La Jolla, CA, USA).

## Results

### WNT ligands expression signatures are tumor-specific

**Fig. 1:**
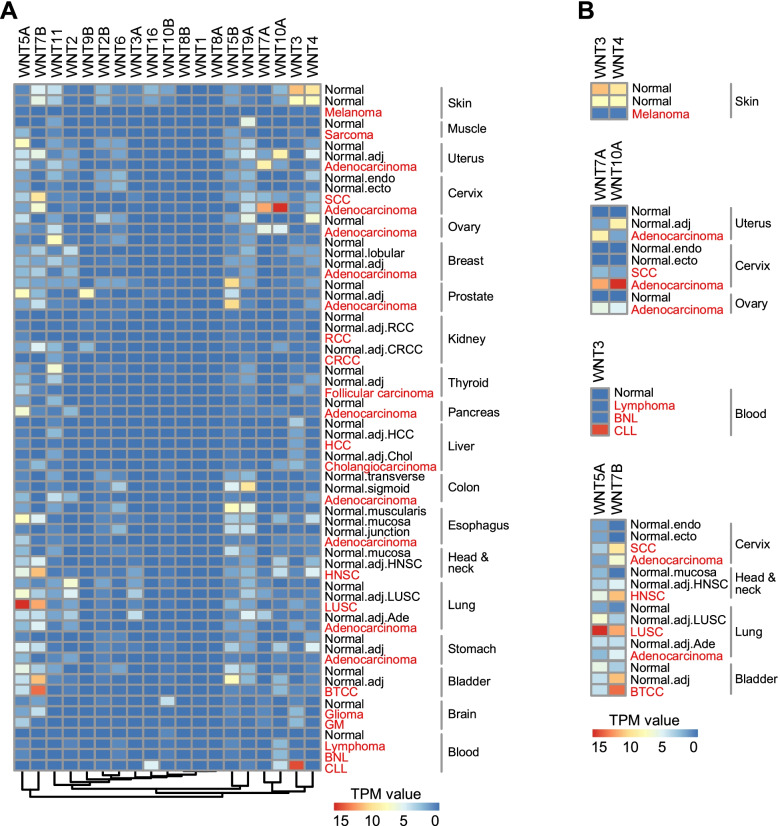
Expression profiling of WNT ligands in distinctive tumors and matched normal tissues. **A** mRNA expression profiling of 19 WNTs in 26 kinds of tumors, matched adjacent normal tissues and human normal tissues (downloaded from Expression Atlas; https://www.ebi.ac.uk/gxa/experiments/E-MTAB-5200). All data used for analysis were normalized to TPM (Transcripts Per Million) and run in Pretty Heatmaps with R package version 3.6.3 (https://github.com/cran/pheatmap). We used the command “cluster_cols = false” to merely cluster genes, and Euclidean distance was used to calculate the distance. **B** Representative sub-clusters of WNT ligands that showed tissue or tumor-specific expression patterns. Two sets of normal skin data referred to the skin from normal leg and suprapubic skin separately. The normal brain data represented the whole brain tissue. SCC: cervical squamous cell carcinoma; RCC: renal cell carcinoma; CRCC: clear renal cell carcinoma; HCC: hepatocellular carcinoma; HNSC: squamous cell carcinoma of head and neck; LUSC: lung squamous cell carcinoma; BTCC: transitional cell carcinoma of bladder; GM: glioblastoma multiforme; BNL: B-cell non-Hodgkin lymphoma; CLL: chronic lymphocytic leukemia

To investigate whether different WNT ligands function uniquely across multiple cancer types, we first compared 19 WNT ligands mRNA expression profiling across distinctive tumors with matched paratumor tissues, as well as normal tissues. The tumor datasets were downloaded from Expression Atlas (https://www.ebi.ac.uk/gxa/experiments/E-MTAB-5200) [13]. The expression of WNTs showed a tissue-dependent and tumor-dependent pattern (Fig. 1A). According to their expression feature, WNTs could be divided into 3 categories, tumor positive, negative, and irrelevant. WNT11, WNT9A and WNT4 were tumor negative WNTs, which were abundant in normal tissues, while declining in many tumors including melanoma, sarcoma, breast adenocarcinoma and colorectal adenocarcinoma. Conversely, WNT5A, WNT7B, WNT2, WNT7A, WNT10A and WNT3 were tumor-positive WNTs, which significantly increased in many carcinomas. The remnants were tumor irrelevant WNTs. A panel of WNTs was suggested to contribute to tissue-specific tumorigenicity. Figure 1B listed several sub-clusters. Among them, WNT3 and WNT4 were implied as melanoma tumor suppressors; WNT7A and WNT10A displayed contribution to the female reproductive system adenocarcinomas. WNT3 and WNT16 specifically increased in chronic lymphocytic leukemia (CLL), indicating the specificity of WNTs in leukemia. And the opposite effects of WNT3 in solid tumors and leukemia accentuated the complexity of WNT signaling in tumors. Surprisingly, WNT5A and WNT7B were significantly upregulated in CSCC, HNSC, LUSC and BTCC, suggesting universal property of being postulated as pan-SCCs biomarkers.

### WNT receptors and co-receptors exhibit histology-dependent tumor specificity

**Fig. 2:**
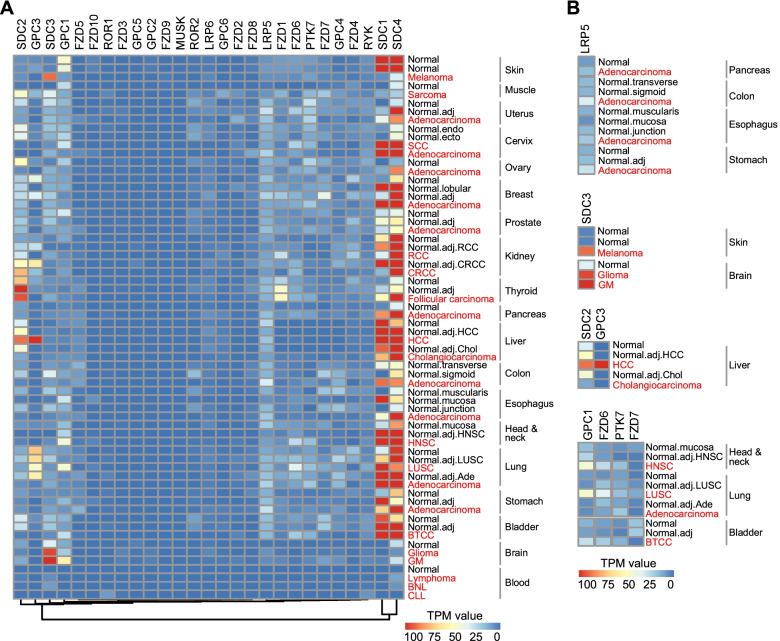
Expression profiling of WNT receptors and co-receptors in distinctive tumors and matched normal tissues. **A** mRNA expression profiling of 27 WNT receptors and co-receptors in 26 kinds of tumors, matched adjacent normal tissues and human normal tissues (downloaded from Expression Atlas; https://www.ebi.ac.uk/gxa/experiments/ E-MTAB-5200). Data were processed with similar analytical methods of WNT ligands. **B** Representative sub-clusters of WNT receptors and co-receptors that showed tissue or tumor-specific expression patterns

WNTs employ more than 15 receptors and co-receptors of different families. These receptors are simultaneously regulated by a variety of antagonists or agonists such as R-spondins [3]. Using similar analytical methods of WNT ligands, we examined the expression profiling of WNT receptors and co-receptors, as well as four syndecans, R-spondins interacting transmembrane proteoglycans, in the above tumors. Among those 27 WNT receptors and co-receptors, FZD5, FZD10, ROR1, FZD3, GPC5, GPC2, FZD9 and MUSK were defined as low abundance genes, TPM of these genes were smaller than 5 in all tissues (Fig. 2A). GPC3, ROR2, and FZD4 were negatively correlated with many malignant tissues (Fig. 2A). While SDC3, FZD2, FZD8, LRP5, LRP6, FZD6, PTK7, GPC4, RYK, SDC1 and SDC4 were positively correlated with many cancers (Fig. 2A). LRP5 and LRP6 act as co-receptors in β-catenin-dependent signaling. LRP6 was negligible in all above tissues compared with LRP5 (Fig. 2A). However, LRP5 preferred adenocarcinomas of digestive organs, including the pancreas, colon, esophagus and stomach (Fig. 2B). SDC3 was specifically increased in melanoma and two brain tumors, glioma and glioblastoma multiforme. SDC2 and GPC3 were up-regulated in HCC. Intriguingly, similar to WNT ligands, the SCCs demonstrated the enrichment of a set of WNT receptors and co-receptors, including GPC1, FZD6, PTK7 and FZD7 (Fig. 2B).

Taken together, these results suggested that specified WNT ligands and receptors/co-receptors could be attributed to distinctive tumors according to their tissue-of-origin.

### Validation of WNTs specificity in oral squamous carcinoma

**Fig. 3:**
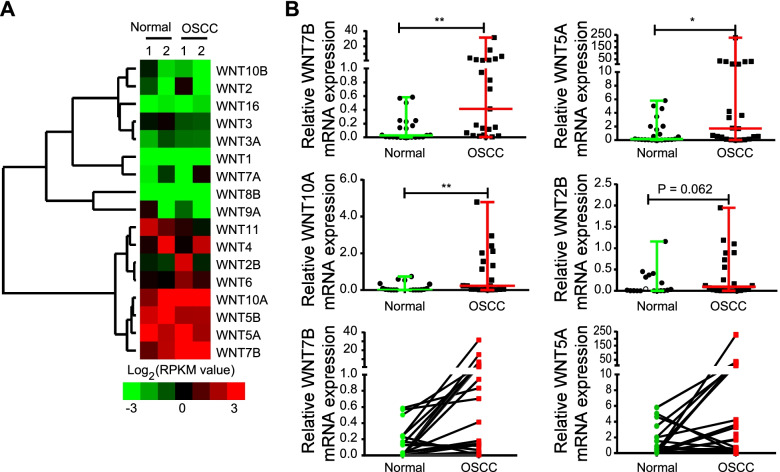
Squamous epithelium cell carcinoma positive WNTs verification in OSCC. **A** WNT ligands expression profiling was revealed by RNA-seq analysis in two OSCC patients (GSE70666) [14]. We used the log2 ratio of normalized expression of mRNAs in samples as inputs, calculated the distance using Euclidean distance, and completed the linkage hierarchical clustering method for clustering [15]. Results were visualized by Java Treeview. **B** qRT-PCR verification the mRNA expression levels of 4 OSCC positive WNTs in adjacent normal and OSCC tissues (Normal: *n* = 23, OSCC: *n* = 23, for WNT7B and WNT5A detection; Normal: *n* = 22, OSCC: *n* = 22, for WNT2B and WNT10A detection). *: *P* < 0.05; **: *P* < 0.01

Given the specific expression pattern of WNT components in SCCs, we initially focused on quantifying WNTs expression in OSCC. Our previous work has compared genome profiling of adjacent normal, premalignant and OSCC tissues collected from two recruited OSCC patients (GSE70666) [14]. Here, we revisited the WNT ligands expression between paired tumors and adjacent normal tissues. Our data annotated 17 WNTs and found that WNT2B, WNT10A, WNT5A and WNT7B showed a similar cancer-positive pattern, and WNT7B was the most significant one (Fig. 3A). Subsequently, we confirmed this result by qRT-PCR in 21 OSCC clinical samples and paired adjacent nontumorous tissues. Consistently, we detected increased mRNA expression of WNT*7B* (*P* = 0.0003), as well as WNT10A (*P* = 0.0023) and WNT5A (*P* = 0.088), in most cancer samples (Fig. 3B). However, WNT2B showed no statistically significant difference (Fig. 3B). Of note, WNT10A mRNA was statistically different between tumor and adjacent normal tissue, but its abundance was low in these samples. Furthermore, WNT5A expression exhibited obvious heterogeneity (Fig. 3B, bottom panel).

### Validation of LUSC specific WNT ligands and receptor/co-receptor reveals a correlation between mortality and expression pattern

**Fig. 4:**
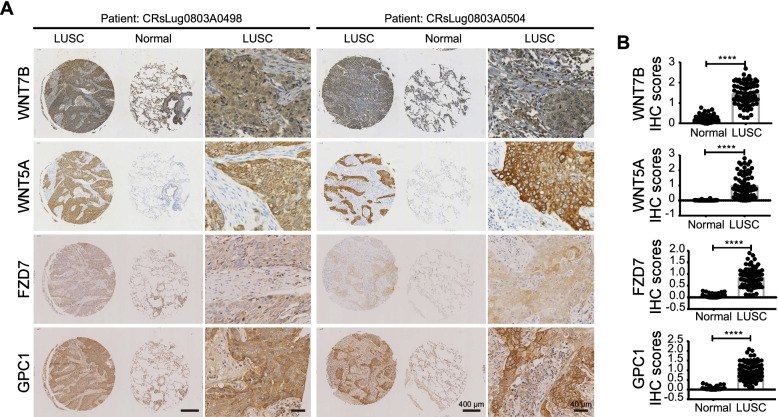
Squamous epithelium cell carcinoma positive WNT ligands and receptors/co-receptors verification in LUSC. **A, B** IHC identification (**A**) and quantitative analysis (**B**) of WNT7B, WNT5A, FZD7 and GPC1 proteins expression in 72 paired adjacent normal and LUSC tissues. (Scale bar = 400 μm or 40 μm separately). ****: *P* < 0.001

To further evaluate the universalism of altered WNT5A and WNT7B in a broad range of SCCs, we examined them by IHC in LUSC tissue chips (Fig. 4A). Simultaneously, we also detected the expression of two SCCs specific WNT receptor and co-receptor, FZD7 and GPC1, in the same samples (Fig. 4A). As shown in Fig. 4B, a statistically significant elevation of WNT7B, WNT5A, FZD7 and GPC1 was observed between 72 LUSC tumors and adjacent normal tissues, consistent with the aforementioned transcriptomic analysis (Figs. 1 and 2). Similar to OSCC, WNT5A expression in LUSC was also heterogeneous as the CV (coefficient of variation) of WNT5A IHC score was 0.79. Instead, for WNT7B, FZD7 and GPC1, the CV was 0.38, 0.48 and 0.47, respectively. While positive staining for WNT7B was observed obviously in the cytoplasm of the majority of malignant tissues, WNT5A, FZD7 and GPC1 signals were presented on both the cytoplasm and membrane of cancer cells (Fig. 4A).

**Fig. 5:**
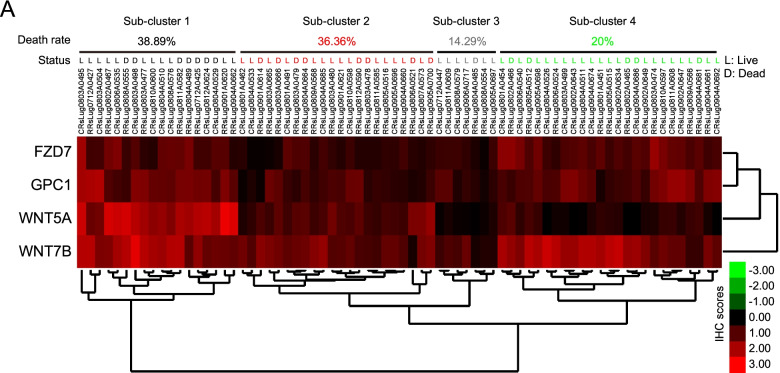
The correlation analysis between mortality and WNT7B, WNT5A, FZD7, GPC1 protein expression patterns. **A** The scores of semi-quantitative IHC were used as inputs, calculated the distance using Euclidean distance, and completed the linkage hierarchical clustering method for clustering. Results were visualized by Java Treeview. 72 cases of patients were divided into 4 subgroups according to protein expression patterns of WNT7B, WNT5A, FZD7, GPC1, and mortality of each subgroup was counted

Next, we examined whether these proteins expression impacts clinical output. Using IHC score in individual LUSC tumor tissue as a reference, we found that the expression profiling of WNT7B, WNT5A, FZD7 and GPC1 could be divided into four sub-clusters (Fig. 5A). We further compared the death rate within each sub-cluster. Sub-cluster 1 showed high expression of all four proteins and exhibited the highest death rate, 38.89% (Fig. 5A). In sub-cluster 2, all proteins were expressed in medium abundance, of which the death rate was 36.36% (Fig. 5A). The lowest death rate was 14.29%, which belonged to sub-cluster 3 characterized by a low abundance of all proteins (Fig. 5A). As for sub-cluster 4, it had highly expressed WNT7B, GPC1 and FZD7, except for WNT5A. And it showed a low death rate, 20% (Fig. 5A). In addition, an metastasis analysis showed that the expression level of the individual gene has no significant effect on HNSC (in TCGA datasets, OSCC samples were brought into HNSC) or LUSC overall survival, except for GPC1 in HNSC, the *p*-value of HR (Hazard Rate) of GPC1 in HNSC is 0.03 (supplementary Fig. 1). These results showed that the combined expression of WNT7B, WNT5A, GPC1 and FZD7 contributed to LUSC malignancy, and the clinical output partly relied on the balance between WNT5A and the other proteins.

### WNT7B is involved in the malignant development of oral inflammation and carcinoma

**Fig. 6:**
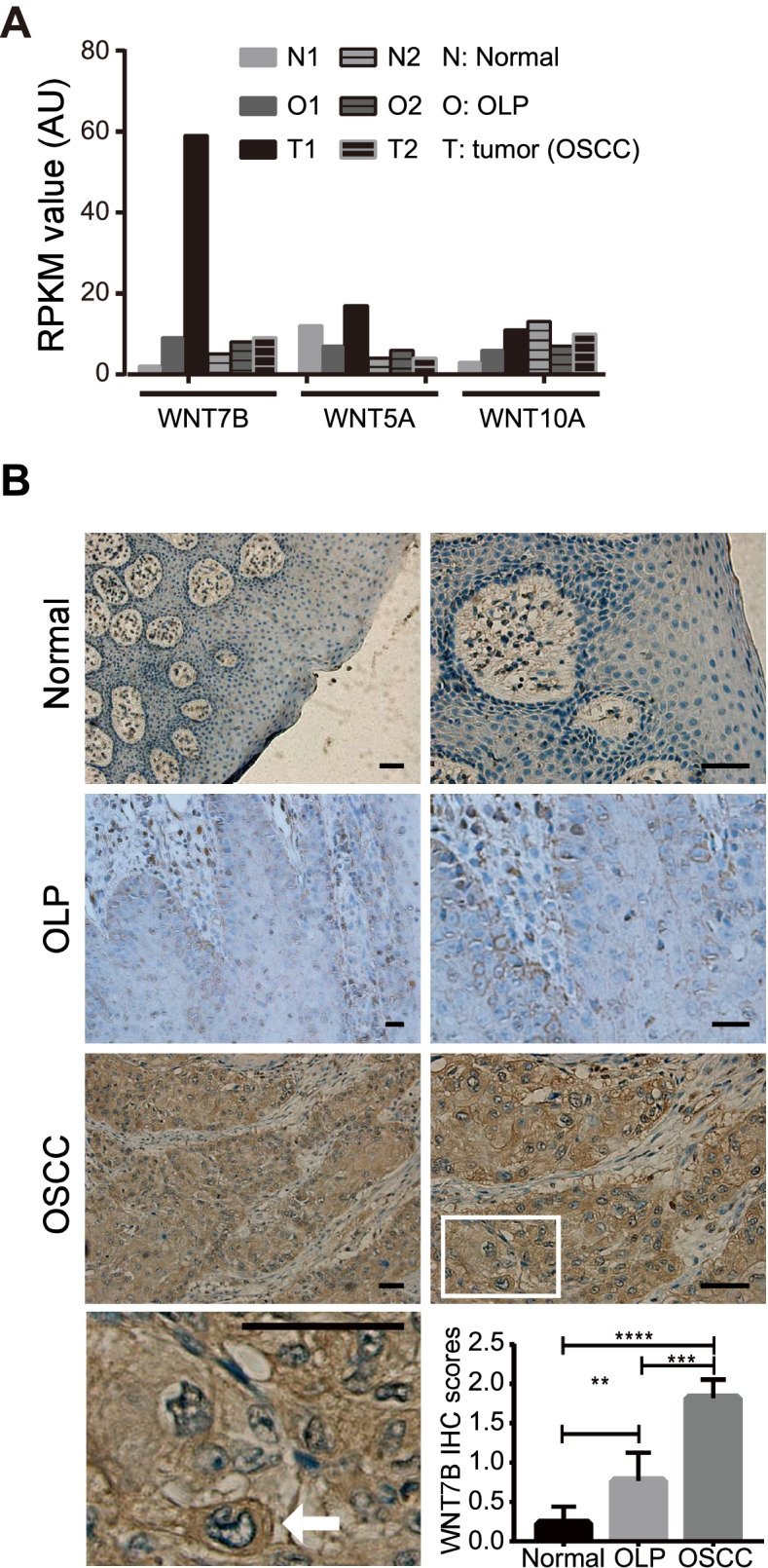
Participation of WNT7B in malignant development of oral inflammation and carcinoma. **A** Three verified OSCC positive WNTs expression profiling revealed by RNA-seq analysis in clinical OLP and OSCC patients (GSE70666). N: adjacent normal tissue; O: OLP tissue; T: OSCC tissue. **B** IHC identification and quantitative analysis of WNT7B expression in clinical OLP and OSCC patients (Scale bar = 100 μm). The white square frame tagged area was magnified (lower left pane), and the white arrowhead indicates macrophage among tumor tissues. **: *P* < 0.01; ***: *P* < 0.005; ****: *P* < 0.001

Generally, there existed a prolonged inflammation course in OSCC and LUSC development [16–18]. Moreover, WNT7B was suggested to participate in chronic inflammations [19]. We found a high expression of WNT7B in the above OSCC and LUSC patients as well (Figs. 1, 3 and 4). Therefore, we assumed that WNT7B may affect oral inflammation and cancer progression. We revisited the mRNA expression of WNT7B, WNT5A, and WNT10A in our prior data (GSE70666) [14]. Among these OSCC positive WNTs, only WNT7B was progressively increased in normal, OLP (oral lichen planus), and OSCC tissues in both patients (Fig. 6A). We further confirmed WNT7B expression using 34 FFPE samples by IHC. Compared with adjacent nontumorous tissues, WNT7B positive signals were gradually increased in OLP, then to OSCC (Fig. 6B). Moreover, positive signals predominantly existed in OLP pathological epithelium and OSCC cancer cells (Fig. 6B). Notably, a strong positive WNT7B signal also existed in macrophages (Fig. 6B). Thus, these results suggested the feasibility of using WNT7B as an indicator of the transition from oral inflammation to tumor progression.

### WNT7B promote tumor invasion by its downstream genes including MMP1

WNT signaling initiates proliferation and EMTs in multiple carcinomas [20]. Considering the gradual up-regulation of WNT7B in OLP and OSCC, we sought to investigate the biological functions of WNT7B in the OSCC progression. We constructed WNT7B stable knockdown OSCC cell lines (SCC9 and FaDu) (Supplementary Fig. 2A) and performed a proliferation assay and cell cycle detection to evaluate the contribution of WNT7B to cell growth. Results showed that the knockdown of WNT7B did not inhibit the proliferation of tumor cells (Supplementary Fig. 2B). However, WNT7B was suggested to improve the tolerance of cells to nutritional deficiency, as WNT7B knockdown cells recovery from cell cycle synchronization more slowly than the control (Supplementary Fig. 2C and D).

**Fig. 7:**
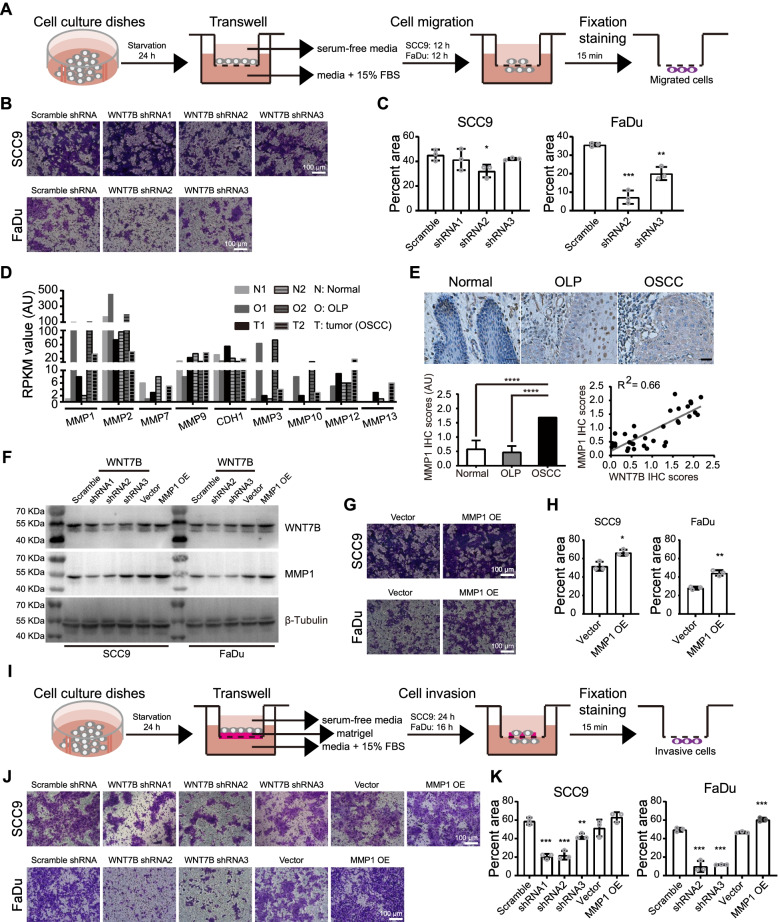
WNT7B knockdown attenuates the invasion ability of OSCC tumors through WNT signaling downstream target MMP1. **A** Schematic illustration of the transwell cell migration assay. **B**, **C** Representative images (**B**) and quantification (**C**) of area covered by migrated cells in wild-type and WNT7B stable knockdown OSCC cell lines. Each measurement was performed in three independent biological replicates. Statistical significance was assessed by unpaired Student’s t test; *: *P* < 0.05; **: *P* < 0.01; ***: *P* < 0.001. **D** WNT signaling downstream and tumor invasion associated MMP expression profiling revealed by RNA-seq analysis in clinical OLP and OSCC patients (GSE70666) [14]. N: adjacent normal tissue; O: OLP tissue; T: OSCC tissue. **E** IHC analysis of MMP1 protein expression in clinical patients and the correlation between WNT7B and MMP1 IHC scores in the same clinical samples (Adjacent normal tissue: *n* = 7; OLP: *n* = 15; OSCC: *n* = 12). Statistical significance was assessed by unpaired Student’s t test; ****: *P* < 0.001. **F** Western blot analyses of WNT7B and MMP1 expression in wild-type and WNT7B stable knockdown OSCC cell lines. Full-length blots are presented in Supplementary Fig. 4. **G**, **H** Representative images (**G**) and quantification (**H**) of area covered by migrated cells in wild-type and MMP1 overexpression (OE) cell lines. Each measurement was performed in three independent biological replicates. Statistical significance was assessed by unpaired Student’s t test; *: *P* < 0.05; **: *P* < 0.01. **I** Schematic illustration of the transwell cell invasion assay. **J**, **K** Representative images (**J**) and quantification (**K**) of area covered by invasive cells in wild-type and WNT7B stable knockdown OSCC cell lines. Each measurement was performed in three independent biological replicates. Statistical significance was assessed by unpaired Student’s t test; **: *P* < 0.01; ***: *P* < 0.001. For detailed numbers and statistical analysis, see Supplementary Table 4

We subsequently examined the effects of WNT7B on the migration of OSCC cells and found WNT7B knockdown had little effect on the capacity of SCC9 cells to migrate, whereas greatly inhibited the migration of FaDu cells (Fig. 7A-C). Given WNT7B is typically involved in canonical WNT signaling, we then investigate whether this invasion capability was attributed to canonical signaling downstream genes. According to the probable WNT target genes (http://web.stanford.edu/group/nusselab/cgi-bin/WNT/target_genes), we chose CDH1 (cadherin1) and matrix metalloproteinases (MMPs) as candidates because they show a high correlation with tumor invasion and metastasis [21, 22]. We compared the mRNA abundance of all WNT downstream MMP members and CDH1 in normal, OLP, and cancer tissues using our previous data (GSE70666) [14]. MMP1, MMP3, and MMP12 were up-regulated in both inflammation and cancer (Fig. 7D). However, MMP1 was the most significant increased one and showed well consistency in two OSCC patients. Further verification of MMP1 expression in the above 34 FFPE samples showed that, contrary to the weak expression in normal tissues, strong cytoplasmic positive signals were accumulated in cancer cells (Fig. 7E). In OLP tissues, the epithelium was loosely organized and a positive signal was localized in epidermal basal cells. Interestingly, FFPE samples showed a good correlation between WNT7B and MMP1 proteins expression (R^2^ = 0.66) (Fig. 7E). Furthermore, compared with the control, knockdown of WNT7B was accompanied by reduced mRNA and protein expression of MMP1 in both SCC9 and FaDu cells (Supplementary Fig. 3 and Fig. 7F). MMP1 overexpression, on the other hand, had minimal influence on WNT7B expression levels in SCC9 and FaDu cells (Fig. 7F). MMP1 overexpression enhanced the migratory ability of these two OSCC cells in a transwell cell migration experiment (Fig. 7G and H). To study the effect of WNT7B on invasion in OSCC cells, we used a transwell cell invasion test (Fig. 7I). We discovered that knocking down of WNT7B reduces the capacity of SCC9 and FaDu cells to invade. Additionally, overexpression of MMP1 had little effect on invasion in SCC9 cells while increasing invasion ability in FaDu cells (Fig. 7J and K). Taken together, our findings showed that WNT7B and MMP1 are involved in oral inflammation and tumor progression.

## Discussion

WNT signaling serves as one of the crucial cellular signaling pathways regulating embryonic development and adult stem cell fate [1, 2]. Recent insights show that mutations of WNT signaling components are causative of multiple cancer types [2, 4]. Considering the diversity of WNT ligands and receptors, we speculated that whether WNT signaling is executed by its different members across distinctive tumors? Do the varied interaction pairs of the WNT ligand and its receptor have a divergent role in cancer?

In this study, we suggest that WNT ligands and receptors could be classified into tumor-promoting or tumor-suppressive according to tissue-of-origin of the tumor. For instance, WNT3 and WNT4 are implied as a tumor suppressor of melanoma (Fig. 1). WNT7A and WNT10A may contribute to female reproductive system adenocarcinomas, while LRP5 prefer adenocarcinomas of digestive organs (Fig. 1). SDC3 is specifically increased in melanoma and brain tumors, but SDC2 and GPC3 are up-regulated in HCC (Fig. 2).

Importantly, our analysis reveals that WNT signaling shows a specific activation pattern in SCCs. Especially, a panel of WNT components, including WNT5A, WNT7B, GPC1, and FZD6 or FZD7, shows tumor tissue-specific increment in CSCC, HNSC, LUSC, and BTCC (Figs. 1 and 2). Although recent evidence has suggested the involvement of some of these genes in HNSC or LUSC [23, 24], for the first time, this work explored the expression profiling of all WNT ligands and receptors in SCCs. Detection using SCC patient samples also supports the above-mentioned conclusions (Figs. 3 and 4), suggesting that this group of WNT members may be utilized as common biomarkers or therapeutic targets of SCCs.

The distinctive types of SCCs are subject to similar environmental risks, especially chronic inflammation and pathogenic virus infection. For example, human papillomavirus (HPV) is a causative agent of SCCs occurred in digestive epithelium [25, 26]. HPV oncoproteins have been suggested to regulate the WNT signaling pathway such as elevating WNT7B mRNA in cervical cancer [27, 28]. Additionally, HPV infected cervical cancer is shown to alter expression of miRNAs targeting WNT5A and FZD6 [29, 30]. Besides, *H. pylori* infection also could activate the WNT pathway via FZD7 [31]. Interestingly, activation of the WNT pathway using microbes is also utilized to propagate intestinal inflammation and control the inflammatory tissue regeneration [32, 33]. For instance, microbes infection stimulates immune cells to secrete inflammatory cytokines, including TNF, IL-6, and IL-17, which simultaneously increase the expression of WNT components such as WNT3A and WNT7B [34]. Furthermore, WNT5A is also demonstrated under the regulation of TNF or IL-17 [35, 36]. Collectively, these indicate that the SCCs specific WNT signaling activation plays a central role in the regulation of chronic inflammation and tumor.

OSCC is among the most widespread and low survival rate HNSC, highly correlated with chronic oral inflammation [16, 37]. WNT components are found to associate with OSCC and the progression of dysplasia in oral leukoplakia [38, 39]. We found that WNT7B is gradually upregulated in OLP and OSCC, and knockdown of WNT7B in OSCC cell lines reduced cell invasive ability (Figs. 6 and 7). Hence, we suggested WNT7B to be a potential molecule mediating OLP and OSCC progression. This result also agrees with earlier work [23]. In addition, WNT7B is required for the angiogenic switch, tumor progression and metastasis in infiltrating myeloid cells [40]. Taken together, these results indicate that WNT7B may serve as a potential regulator for the development of chronic inflammation and OSCCs.

This work provides a new opinion to understand how intricate cellular signaling pathways contribute to complex human diseases, e.g. cancers, and offers a rationale for the exploration of universal pharmacologic gene targets for tumors.

## Supplementary Information

**Additional file 1.**

**Additional file 2: Supplementary Table 1.** Clinical parameters of LUSC patients. **Supplementary Table 2.** Primers for qRT-PCR. **Supplementary Table 3.** Sequences of WNT7B shRNA and Scramble shRNA. **Supplementary Table 4.** Statistical data for all experiments.

**Additional file 3: Appendix A.** Supplementary data

## Data Availability

The transcriptomics data sets of tumors, matched adjacent normal tissues and human normal tissues were downloaded from Expression Atlas (https://www.ebi.ac.uk/gxa/experiments/E-MTAB-5200). The transcriptomics data sets of OSCC and OLP were deposited at GEO (GSE70666; http://www.ncbi.nlm.nih.gov/geo/query/acc.cgi?acc=GSE70666). All other data supporting the findings of this study are included in this manuscript and its supplementary information files, including supplementary data, figures and tables.
