# Supplementary material for: Tumor specificity of WNT ligands and receptors reveals universal squamous cell carcinoma oncogenes
Source: BMC Cancer. 2022 Jul 19;22:790. doi: 10.1186/s12885-022-09898-2 (PMC9295300; doi:10.1186/s12885-022-09898-2)
Supplement: Supplementary file 2 — Additional file 2: Supplementary Table 1. Clinical parameters of LUSC patients. Supplementary Table 2. Primers for qRT-PCR. Supplementary Table 3. Sequences of WNT7B shRNA and Scramble shRNA. Supplementary Table 4. Statistical data for all experiments. [file 12885_2022_9898_MOESM2_ESM.doc]

**Tumor specificity of WNT ligands and receptors reveals universal squamous cell carcinoma oncogenes**

Cheng Chen^1,2#^, Lunan Luo^1,2#^, Changling Xu^1,2#^, Xia Yang^1,2^, Ting Liu^1,2^, Jingyue Luo^1,2^, Wen Shi^3^, Lu Yang^1,2^, Yi Zheng^1,2*^, Jing Yang^1,2*^

1. Department of Health Management, Sichuan Provincial People's Hospital, University of Electronic Science and Technology of China, Chengdu 610054, China
2. School of Medicine, University of Electronic Science and Technology of China, Chengdu 610054, China
3. School of Stomatology, Peking University, Beijing 100191, China

^#^ These authors contributed equally to this work.

^*^Correspondence: [yi_zheng@uestc.edu.cn](mailto:yi_zheng@uestc.edu.cn); [yangjing1977@uestc.edu.cn](mailto:yangjing1977@uestc.edu.cn)

**Supplementary Tables**

Supplementary Table 1. Clinical parameters of LUSC patients.

Supplementary Table 2. Primers for qRT-PCR.

Supplementary Table 3. Sequences of WNT7B shRNA and Scramble shRNA

Supplementary Table 4. Statistical data for all experiments.

**Supplementary Table 1.** **Clinical parameters of LUSC patients.**

| Received surgery date:  2007.11-2009.05  Last follow-up date: 2012.07 | | Male | | Female | |
| --- | --- | --- | --- | --- | --- |
|  |  | Alive | Dead | Alive | Dead |
| Number of patients | | 46 | 20 | 5 | 1 |
| Average age (years) | | 74 | 66 | 57.4 | 48 |
| Distant metastasis of tumor | | no | no | no | no |
| Average tumor volume (cm^3^) | | 96.15 | 98.06 | 76.5 | 49 |
| Lymph node metastasia (patient number & percentage) | | 13 (28.3%) | 8 (40%) | 3 (60%) | 1 (100%) |
| AJCC cancer staging (7_th_ edition) | I | 18 (39.1%) | 6 (30%) | 2 (40%) | 0 |
|  | I-II | 1 (2.2%) | 0 | 0 | 0 |
|  | II | 17 (37%) | 7 (35%) | 0 | 0 |
|  | II-III | 7 (15.2%) | 0 | 3 (60%) | 0 |
|  | III | 3 (6.5%) | 7 (35%) | 0 | 1 (100%) |

**Supplementary Table 2.** **Primers for qRT-PCR.**

| Gene | Forward primer (5’→ 3’) | Reverse primer (5’→ 3’) |
| --- | --- | --- |
| human WNT7B | CTTTGGCGTCCTGTACGTG | CTAGGCCAGGAATCTTGTTGC |
|
| human WNT5A | GAAATGCGTGTTGGGTTGAA | ATGCCCTCTCCACAAAGTGAA |
|
| human WNT2B | CCTGTAGCCAGGGTGAACTG | CGGGCATCCTTAAGCCTCTT |
|
| Human WNT10A | AAGCCTGGAGACTCGCAAC | CAAAAGCGCTCTCTCGGAAAC |
|
| human MMP1 | AGCCATCACTTACCTTGCACT | CTGGGAAGCTGTGAGACACC |
| human ACTIN | CTGAGCGTGGCTACTCCTTC | GCCATCTCGTTCTCGAAGTC |
|
| human GAPDH | ACAACTTTGGTATCGTGGAAGGAC | CAGGGATGATGTTCTGGAGAGC |

**Supplementary Table 3. Sequences of WNT7B shRNA and Scramble shRNA.**

| Vector | Inserted sequence (5’→ 3’) | Target sequence (5’→ 3’) |
| --- | --- | --- |
| pLV-WNT7B-shRNA1 | GTCGGGAGATCAAGAAGAACTTCAAGAGAGTTCTTCTTGATCTCCCGATTTTTT | TCGGGAGATCAAGAAGAAC |
|
| pLV-WNT7B-shRNA2 | GACAGACCTGGTGTACATTTTCAAGAGAAATGTACACCAGGTCTGTCTTTTTTg | GACAGACCTGGTGTACATT |
|
| pLV-WNT7B-shRNA3 | GCCTCATGAACCTGCATAATTCAAGAGATTATGCAGGTTCATGAGGCTTTTTTg | GCCTCATGAACCTGCATAA |
|
| pLV-Scramble-shRNA | ATCGACTAGCCACTTAGACTTCAAGAGGTCTAAGTGGCTAGTCGATTTTTTTT | ATCGACTAGCCACTTAGAC |
|

**Supplementary Table 4. Statistical data for all experiments.**

| **Figure 3** | | | |
| --- | --- | --- | --- |
| **Fig. 3B** | WNT7B | Normal | 0.0304 ± 0.1883 for Normal, n = 23 |
|  |  | OSCC | 0.4137 ± 7.0302 for OSCC, n = 23, *p =* 0.0008, compared to Normal |
|  | WNT5A | Normal | 0.1426 ± 1.8087 for Normal, n = 23 |
|  |  | OSCC | 1.697 ± 47.311 for OSCC, n = 23, *p =* 0.0287, compared to Normal |
|  | WNT10A | Normal | 0.0086 ± 0.2248 for Normal, n = 22 |
|  |  | OSCC | 0.231 ±1.2718 for OSCC, n = 22, *p =* 0.0017, compared to Normal |
|  | WNT2B | Normal | 0.0103 ± 0.2731 for Normal, n = 22 |
|  |  | OSCC | 0.0968 ± 0.5327 for OSCC, n = 22, *p =*0.0616, compared to Normal |
|  | WNT7B | Normal | 0.0304 ± 0.1883 for Normal, n = 23 |
|  |  | OSCC | 0.4137 ± 7.0302 for OSCC, n = 23, *p =* 0.0008, compared to Normal |
|  | WNT5A | Normal | 0.1426 ± 1.8087 for Normal, n = 23 |
|  |  | OSCC | 1.697 ± 47.311 for OSCC, n = 23, *p =* 0.0287, compared to Normal |
| **Figure 4** | | | |
| **Fig. 4B** | WNT7B | Normal | 0.179 ± 0.0208 for Normal, n = 72 |
|  |  | LUSC | 1.444 ± 0.0651 for LUSC, n = 72, *p <* 0.0001, compared to Normal |
|  | WNT5A | Normal | 0.016 ± 0.003 for Normal, n = 72 |
|  |  | LUSC | 0.9578 ± 0.0897 for LUSC, n = 72, *p <* 0.0001, compared to Normal |
|  | FZD7 | Normal | 0.0547 ± 0.008 for Normal, n = 72 |
|  |  | LUSC | 0.8341 ± 0.047 for LUSC, n = 72, *p <* 0.0001, compared to Normal |
|  | GPC1 | Normal | 0.0576 ± 0.009 for Normal, n = 72 |
|  |  | LUSC | 0.9778 ± 0.054 for LUSC, n = 72, *p <*0.0001, compared to Normal |
| **Fig. 6** | | | |
| **Fig. 6B** | WNT7B | Normal | 0.2257 ± 0.0803 for Normal, n = 7 |
|  |  | OLP | 0.766 ± 0.093 for OLP, n = 15, *p =* 0.0016, compared to Normal |
|  |  | OSCC | 1.82 ± 0.068 for OSCC, n = 12, *p <* 0.0001, compared to Normal |
| **Fig. 7** | | | |
| **Fig. 7C** | SCC9 | Scramble  shRNA | 45.1572 ± 3.635 for Scramble, n = 3 |
|  |  | WNT7B shRNA1 | 41.5474 ± 7.0893 for WNT7B shRNA1, n = 3, *p =* 0.5565, compared to Scramble |
|  |  | WNT7B shRNA2 | 32.1529 ± 4.2054 for WNT7B shRNA2, n = 3, *p =* 0.0297, compared to Scramble |
|  |  | WNT7B shRNA3 | 41.9578 ± 0.7755 for WNT7B shRNA3, n = 3, *p =* 0.2904, compared to Scramble |
|  | FaDu | Scramble  shRNA | 35.7021 ± 0.7637 for Scramble, n = 3 |
|  |  | WNT7B shRNA2 | 7.295149 ± 2.94 for WNT7B shRNA2, n = 3, *p* = 0.0002, compared to Scramble |
|  |  | WNT7B shRNA3 | 20.1442 ± 2.8962 for WNT7B shRNA3, n = 3, *p =* 0.0018, compared to Scramble |
| **Fig. 7E** | MMP1  MMP1 | Normal | 0.574 ± 0.117 for Normal, n = 7 |
|  |  | OLP | 0.464 ± 0.060 for OLP, n = 14, *p =* 0.3587, compared to Normal |
|  |  | OSCC | 1.684 ± 0.0095 for OSCC, n = 12, *p* <0.0001, compared to Normal |
| **Fig. 7****H** | SCC9 | Vector | 51.7951 ± 3.9327 for Vector, n = 3 |
|  |  | MMP1 OE | 66.4019 ± 2.6347 for MMP1 OE, n = 3, *p* = 0.012, compared to Vector |
|  | FaDu | Vector | 28.0744 ± 1.2865 for Vector, n = 3 |
|  |  | MMP1 OE | 44.3814 ± 2.4179 for MMP1 OE, n = 3, *p* = 0.0011, compared to Vector |
| **Fig. 7K** | SCC9 | Scramble  shRNA | 58.9439 ± 3.075 for Scramble, n = 3 |
|  |  | WNT7B shRNA1 | 20.7017 ± 2.3176 for WNT7B shRNA1, n = 3, *p =* 0.0001, compared to Scramble |
|  |  | WNT7B shRNA2 | 21.9753 ± 3.8911 for WNT7B shRNA2, n = 3, *p =* 0.0005, compared to Scramble |
|  |  | WNT7B shRNA3 | 42.5751 ± 2.3681 for WNT7B shRNA3, n = 3, *p =* 0.004, compared to Scramble |
|  |  | Vector | 51.5042 ± 7.4106 for Vector, n = 3 |
|  |  | MMP1 OE | 63.2951 ± 4.3349 for MMP1 OE, n = 3, *p* = 0.1241, compared to Vector |
|  | FaDu | Scramble  shRNA | 49.6183 ± 1.7078 for Scramble, n = 3 |
|  |  | WNT7B shRNA2 | 9.887 ± 4.8185 for WNT7B shRNA2, n = 3, *p =* 0.0004, compared to Scramble |
|  |  | WNT7B shRNA3 | 11.7117 ± 0.1959 for WNT7B shRNA3, n = 3, *p <* 0.0001, compared to Scramble |
|  |  | Vector | 46.6225 ± 0.7691 for Vector, n = 3 |
|  |  | MMP1 OE | 60.543 ± 1.5668 for MMP1 OE, n = 3 , *p* = 0.0004, compared to Vector |
| **Supplementary Fig. S2** | WNT7B (SCC9) | Scramble  shRNA | 1 ± 0.122 for Scramble, n = 6 |
|  |  | WNT7B shRNA1 | 0.4917 ± 0.0402 for WNT7B shRNA1, n = 6, *p <* 0.0001, compared to Scramble |
|  |  | WNT7B shRNA2 | 0.4653 ± 0.0463 for WNT7B shRNA2, n = 6, *p <* 0.0001, compared to Scramble |
|  |  | WNT7B shRNA3 | 0.4151 ± 0.0309 for WNT7B shRNA4, n = 6, *p <* 0.0001, compared to Scramble |
|  | WNT7B (FaDu) | Scramble  shRNA | 1.0 ± 0.065 for Scramble, n = 6 |
|  |  | WNT7B shRNA2 | 0.63 ± 0.0988 for WNT7B shRNA2, n = 6, *p <* 0.0001, compared to Scramble |
|  |  | WNT7B shRNA3 | 0.5761 ± 0.1296 for WNT7B shRNA3, n = 6, *<* 0.0001, compared to Scramble |
| **Supplementary Fig. S3** | MMP1 (SCC9) | Scramble  shRNA | 1.0 ± 0.1121 for Scramble, n = 5 |
|  |  | WNT7B shRNA1 | 0.3765 ± 0.1468 for WNT7B shRNA1, n = 6, *p <* 0.0001, compared to Scramble |
|  |  | WNT7B shRNA2 | 0.4189 ± 0.062 for WNT7B shRNA2, n = 6, *p <* 0.0001, compared to Scramble |
|  |  | WNT7B shRNA3 | 0.387 ± 0.0218 for WNT7B shRNA3, n = 6, *p <* 0.0001, compared to Scramble |
|  | MMP1 (FaDu) | Scramble  shRNA | 1.0 ± 0.0553 for Scramble, n = 6 |
|  |  | WNT7B shRNA2 | 0.4913 ± 0.0684 for WNT7B shRNA2, n = 6, *p <* 0.0001, compared to Scramble |
|  |  | WNT7B shRNA3 | 0.5107 ± 0.1007 for WNT7B shRNA3, n = 6, *p < 0.0001*, compared to Scramble |
