# Supplementary material for: Tumor specificity of WNT ligands and receptors reveals universal squamous cell carcinoma oncogenes
Source: BMC Cancer. 2022 Jul 19;22:790. doi: 10.1186/s12885-022-09898-2 (PMC9295300; doi:10.1186/s12885-022-09898-2)
Supplement: Supplementary file 3 — Additional file 3: Appendix A. Supplementary data [file 12885_2022_9898_MOESM3_ESM.docx]

**Appendix A.** **Supplementary data**

**
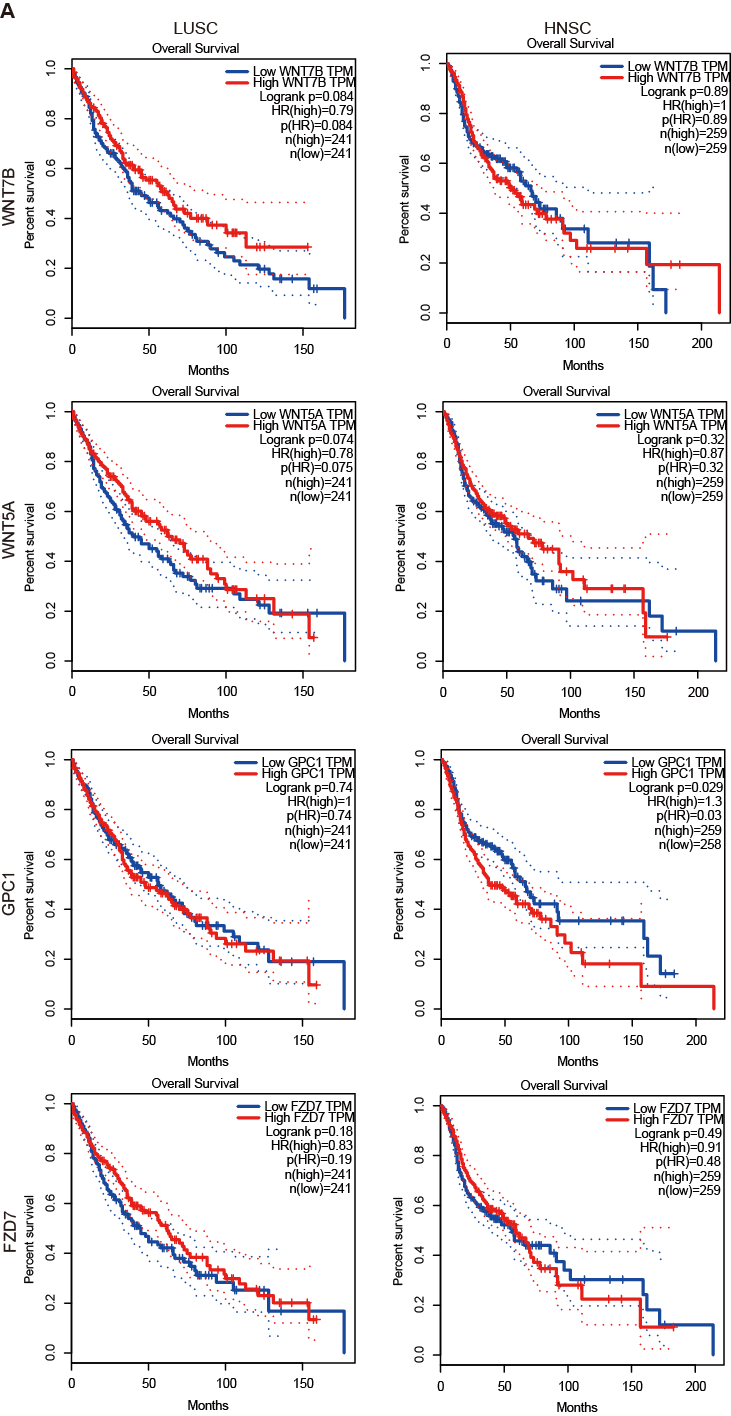
**

Supplementary Fig. 1. The relevance of WNT7B, WNT5A, GPC1 and FZD7 expression levels relate to overall survival rates in LUSC and OSCC.

(A) Analysis of overall survival rates in LUSC and HNSC including OSCC. This metastasis dataset was downloaded from GEPIA (<http://gepia.cancer-pku.cn/>).

**
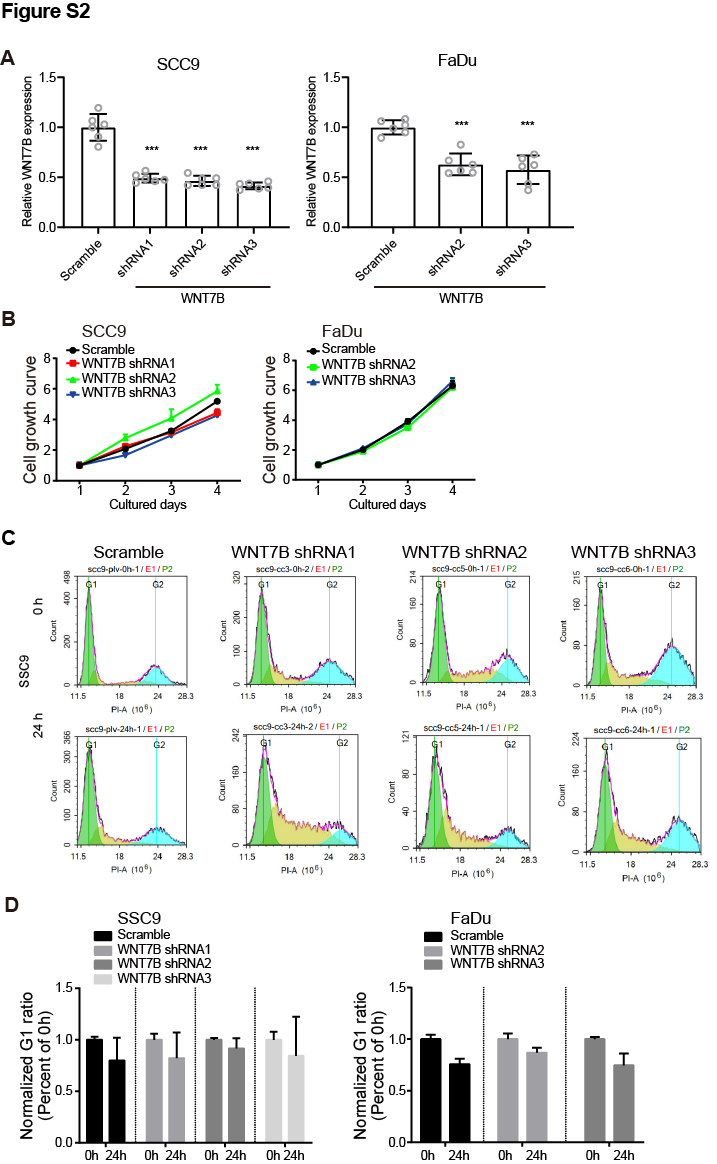
**

Supplementary Fig. 2. Effects of WNT7B knockdown on OSCC cell proliferation and cell cycle.

(A) Quantification of WNT7B mRNA expression in WNT7B knockdown OSCC cell lines using qRT-PCR. Each measurement was performed in three independent biological replicates, and each biological replicate had two duplicated wells. Statistical significance was assessed by unpaired Student’s t test; ***: P < 0.001. (B) Detection of cell proliferation by CCK8 assay for the indicated OSCC cell lines and days. (C) Cell cycle detection by flow cytometry of wild-type and WNT7B stable knockdown OSCC cells after cell cycle synchronization. The cells released after cell cycle synchronization at 0 or 24 hours were used for experiments. (D) Normalized G1 ratio of indicated cell lines. Each measurement was performed in three biological replicates.


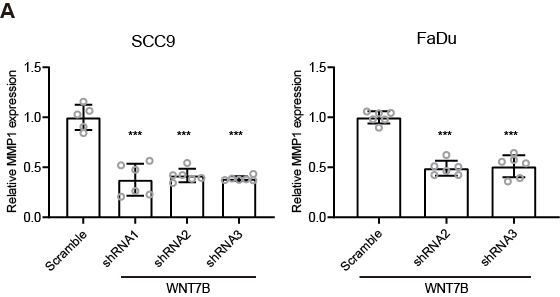


Supplementary Fig. 3. Quantification of MMP1 mRNA expression in WNT7B knockdown OSCC cell lines.

(A) The MMP1 mRNA expression levels were detected by qRT-PCR in indicated OSCC cell lines. Each measurement was performed in three independent biological replicates, and each biological replicate had two duplicated wells. Statistical significance was assessed by unpaired Student’s t test; ***: P < 0.001.


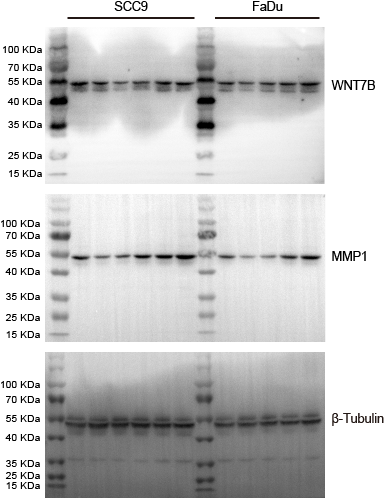


Supplementary Fig. 4. Full-length uncropped blots for Fig. 7F.
